# Analytical method to derive environmental policy effects in an endogenous growth model with leisure

**DOI:** 10.1016/j.mex.2022.101840

**Published:** 2022-09-13

**Authors:** Yoshihiro Hamaguchi, Miraj Ahmed Bhuiyan, Muhammad Khalilur Rahman

**Affiliations:** a Department of Management Information, Kyoto College of Economics, 3-1 Oehigashinagacho, Kyoto, Nishikyo-ku 610-1195, Japan; b School of Economics, Guangdong University of Finance & Economics, 21 Lun Tou Lu, Haizhu District, Guangzhou, Guangdong Province 510320, China; c Faculty of Entrepreneurship and Business, and Angkasa-Umk Research Academy, Universiti Malaysia Kelantan, Kota Bharu, Malaysia

**Keywords:** Pollution permits, Innovation, Growth effect, Welfare effect, Stability analysis

## Abstract

There is deep-rooted opposition to strict environmental regulations, stating that they will lead to job losses and production contraction. Identifying environmental policies compatible with economic growth and pollution reduction is necessary to promote sustainable development. Using an R&D-based model with an endogenous labor supply, we examine the positive effect of an environmental policy on economic growth and welfare, where the policy reduces pollution emissions. The results show a substitution effect, where a reduction in pollution permit levels causes households to substitute labor for leisure and move their labor from production to R&D activities. This policy increased consumption. Thus, reducing pollution permit levels increases the growth rate and welfare via the substitution effect. This methodology can be applied to facilitate the complete analysis of environmental policy effects in an R&D-based growth model. Additionally, applying this analytical approach to other endogenous growth models and simulation analyzes can reveal the mechanisms of various environmental policy effects. In summary, this method facilitates the following steps:

• Analysis of growth and welfare effects of environmental policies.
• Understanding the process of deriving these effects in a basic R&D-based growth model.
• A framework that can be applied to the simulation analysis of these effects was provided.

## Graphical abstract

**Figure:**
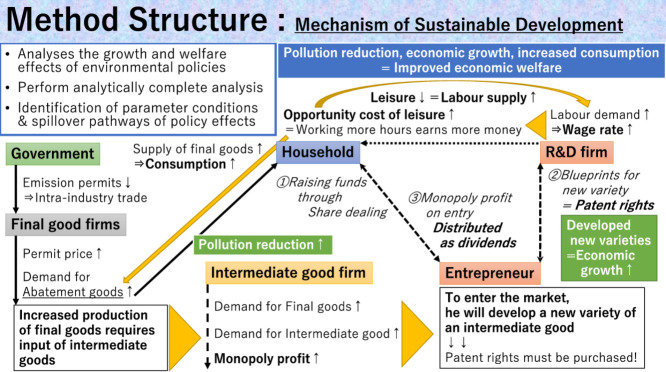

Specifications table

**Table:** 

Subject area:	Economics and Finance
More specific subject area:	Environmental Economics, Macroeconomics
Name of your method:	Dynamic general equilibrium analysis
Name and reference of the original method:	Y. Hamaguchi, Environmental policy effects: an R&D-based economic growth model with endogenous labor supply, J. Econ. Policy Reform 24 (2) (2021) 236-252. https://doi.org/10.1080/17487870.2019.1631598 Y. Hamaguchi, Positive effect of pollution permits in a variety expansion model with social status preference, Manch. Sch. 87(4) (2019) 591-606. https://doi.org/10.1111/manc.12270 Y. Hamaguchi, Environmental policy and social status preference for education in an Uzawa–Lucas model, Bull. Econ. Res. 73(3) (2021) 456-468. https://doi.org/10.1111/boer.12259
Resource availability:	None.

## Method details

### Introduction

As the climate crisis becomes more apparent, the aim is to achieve sustainable development by balancing economic growth with pollution reduction. To achieve this, governments must introduce stringent environmental policies. However, these policies may increase unemployment through the stagnation of productive activities. Recent empirical studies support this argument [1]. Environmental taxes, a typical environmental policy, are considered to have ambiguous effects on economic growth rates and may not necessarily contribute to economic growth ([2,3]). In particular, the empirical analysis in [4] found that in countries with low initial levels of GDP per capita, environment-related tax revenues lead to lower rates of economic growth, whereas in countries with high initial levels of GDP per capita, such tax revenues lead to higher rates of growth.

Political conflicts over environmental issues may arise if political arguments for environmental deregulation intensify job retention and poverty alleviation prioritising job retention over sustainable development. These arguments suggest that the growth-enhancing effects of environmental policies depend on certain conditions. In this case, environmental policies promoting economic growth in one country may lower the rate of economic growth when introduced in another. The key here is to identify the mechanisms through which environmental policies bring about sustainable development and to determine the factors influencing their growth-enhancing effects.

Pioneering theoretical analyzes have, therefore, concentrated on determining the mechanism (e.g. [5], [6], [7], [8], [9], [10]). These studies find an externality effect: the quality of the environment, which is improved by pollution reduction, increases firms' productivity, thereby leading to production expansion and encouraging the accumulation of physical capital through investment. Alternatively, it encourages the proliferation of human capital by improving education productivity. Hence, pollution reduction through environmental policies will bring about sustainable development through this externality effect. Ricci surveyed other relevant studies in [11]. According to this classification, it is essential to identify another channel that does not depend on externality effects. This is because the externality effect assumes a society in which the quality of the environment significantly impacts the economy, which may be limited to a highly industrialised society.

Several empirical case studies have found that increased pollution emissions reduce household labor supply ([12,13]). An important implication here is the impact of pollution on the labor supply. Suppose that strict environmental policies increase labor supply through pollution reduction, which is then used to fund productive activities that promote economic growth, such as firms' R&D activities. In this case, environmental policies can achieve sustainable development without relying on an external effect. This is the focus of the present study.

Hamaguchi studied this issue in [15] using the basic R&D-based growth model in [14]. This study draws on previous studies that have analyzed the effects of environmental policies on economic growth through leisure, namely, the Uzawa-Lucas model in [16] and the learning effects model in [17]. However, these studies are incomplete and do not fully identify the parameter areas in which environmental policies lead to sustainable development. Identifying this parameter area through a complete analysis would help determine which environmental policies of economies lead to sustainable development. Furthermore, this identification will increase simulation and forecasting accuracy in [18], where a comparative dynamic analysis was performed using numerical analysis in [16].

Finally, as the study in [19] points out, environmental taxes in the studies of [16], [17], [18] suffer from the technical modelling problem in that environmental tax rates diverge to infinity in the infinite period ahead. In addition, these previous studies chose environmental taxes as a policy instrument and did not consider emissions trading, which is another typical environmental policy. Considering emissions trading would solve this technical problem and simultaneously demonstrate that it is possible to promote sustainable development without relying on a specific policy instrument. Thus, the main contribution of this study is not only to show that environmental policy has a growth-promoting effect, even when R&D activities are a source of economic growth, but also to refine analytical methods for the growth effects of environmental policy in endogenous growth models that include leisure.

The method in this study presents a way to interpret the channels through which environmental policies lead to sustainable development based on the derivation of the full analytical solution and its parameter conditions from [15], which remains a numerical analysis. Here, a complete analytical study in [19,20] indicates that the social status preferences analyzed in [21] play an important role in giving the growth-enhancing effect to environmental policies. Thus, this study's method can comprehensively analyze environmental policy effects using these analytical methods. Previous studies have identified the growth-promoting effects of environmental policies, independent of an externality effect. For example, there is a generation turnover effect in the continuous overlapping generations model in [22], profit effects in various expansion models without capital in [23], and a public goods model in [24]. However, most studies have not specified the parameter conditions for their growth effects. The analytical approach used in this study may provide useful insights into the search for unknown growth-promoting effects of environmental policies.

### The model

The economy consists of a representative household, a final goods sector, an intermediate goods sector, and an R&D sector. Perfect competition prevails in the final goods sector, which employs labor and intermediate goods to produce the final goods. Using capital stock generates pollution flow and using an abatement good produced from the final good can reduce pollution flow. The intermediate goods firm rents capital from the household and uses capital to produce the intermediate goods. Perfect competition prevails in the R&D sector, which employs labor to produce new designs. The number of households and the population were normalised to one. Households live infinitely in the economy and supply labor. Households acquire positive utility from consumption and leisure and suffer from the negative externalities of pollution.

### Final good sector

Following [5], we assume the following mechanism produces net pollution flow:

(1) $$P_{t}=\frac{\int_{0}^{A_{t}}x_{j,t}dj}{Z_{t}},$$

where $\int_{0}^{A_{t}}x_{j,t}dj\equiv K_{t}$, $Z_{t}$, $x_{j,t}$, and $A_{t}$ represent the aggregate stock of physical capital, abatement good, quantity of intermediate goods j, and number of intermediate goods, respectively. Using the aggregate stock of physical capital increases the net pollution flow, whereas using abatement goods as an end-of-pipe technology decreases the flow.

The government opens the market for pollution permit to internalize negative environmental externalities and allocates pollution permits ($\bar{P}$) to firms in each period. Firms can freely trade allocated pollution permits in a competitive pollution permit market, where, $p_{t}^{e}$ represents the unit price of the pollution permit. The pollutant firms with emissions above the pollution permit $\left(P_{t}>\bar{P}\right)$ must purchase the pollution permit of $\left(P_{t}-\bar{P}>0\right)$ in the market at the price $p_{t}^{e}$, while the abatement firms with emissions under the allocated pollution permit ($P_{t}<\bar{P}$) can sell the pollution permit of ($\bar{P}-P_{t}>0$) in the market at price $p_{t}^{e}$. Thus, pollution permit market must be cleared.

The production function of the final good becomes:

(2) $$Y_{t}=L_{Y,t}^{1-\alpha }\int_{0}^{A_{t}}x_{j,t}^{\alpha }dj,$$

where $Y_{t}$ is the output of the final good, which we consider s numeraire good. Then, $L_{Y,t}$ and 0<α<1 are labor in the final good sector and the ratio of expenditures on intermediate goods to total expenditures for input requirements, respectively. Taking the factor prices as given, the final goods firms choose their inputs to maximize the following production function:

(3) $$\max_{L_{Y,t},x_{j,t},Z_{t}}\Pi_{t}=Y_{t}-w_{t}L_{Y,t}-\int_{0}^{A_{t}}p_{j,t}x_{j,t}dj-Z_{t}-p_{t}^{e}\left(P_{t}-\bar{P}\right),$$

where $w_{t}$, $p_{j,t}$, $p_{t}^{e}$, and $\bar{P}$ represent the wage rate in the final goods sector, the price of intermediate goods j, the price of the pollution permit, and the allocated pollution permit for the firm in each period, respectively. The first-order conditions of profit maximization are given by:

(4) $$w_{t}=\left(1-\alpha \right)\frac{\int_{0}^{A_{t}}x_{j,t}^{\alpha }dj}{L_{Y,t}^{\alpha }},$$

(5) $$p_{j,t}+\frac{p_{t}^{e}}{Z_{t}}=\alpha {\left(\frac{L_{Y,t}}{x_{j,t}}\right)}^{1-\alpha },$$

(6a) $$1=\frac{p_{t}^{e}}{Z_{t}}\frac{\int_{0}^{A_{t}}x_{j,t}dj}{Z_{t}},$$

Where (4)–(6) states that a firm employs labor, intermediate good j, and abatement good, respectively, until their marginal products equal their factor prices.

### Intermediate good sector

Each intermediate goods firm operates as a monopoly firm. Firms pay fixed-cost investments in purchase designs developed by the R&D sector and hold patents. Firms maximize their profits by taking the inverse demand function for their intermediate goods. The variable costs are interest costs. Thus, firms maximise the following:

$$\max_{x_{j,t}}\pi_{j,t}=p_{j,t}x_{j,t}-r_{t}x_{j,t},\;s.t\;p_{j,t}=\alpha {\left(\frac{L_{Y,t}}{x_{j,t}}\right)}^{1-\alpha }-\left(\frac{1}{P_{t}}\right)$$

The first-order conditions for profit maximization are:

(6b) $$p_{t}=\frac{1}{\alpha }\left(r_{t}+\frac{1-\alpha }{P_{t}}\right),$$

(7) $$\pi_{t}=\frac{1-\alpha }{\alpha }\left(r_{t}+\frac{1}{P_{t}}\right)x_{t}.$$

Substituting price into the inverse demand function for intermediate good j determines the quantity of intermediate goods x. Hence, all intermediate goods firms' prices and output levels are equal.

### R&D sector

The following technology develops a new variety of intermediate goods:

(8) $${\dot{A}}_{t}=\delta A_{t}L_{A,t},$$

where $A_{t}$, $L_{A,t}$, $P_{A_{t}}$, and δ represent the stock of the variety's intermediate good, labor in the R&D sector, a parameter of productivity, and the price of a new design, respectively. Perfect competition prevails in the R&D sector and so free entry into the sector results in the following:

(9) $$\delta P_{A,t}A_{t}=w_{t}.$$

### Household

The representative household maximizes the following lifetime utility function:

(10) $$U_{t}=\int_{0}^{\infty }\left[\beta \log C_{t}+\left(1-\beta \right)\log l_{t}-\eta_{P}\log P_{t}\right]e^{-\rho t}dt,$$

where $C_{t}$, $l_{t}$, 0<β<1, $\eta_{p}$, and ρ represent consumption, leisure, weight on the utility attached to consumption and leisure, weight on the utility attached to pollution, and the subjective rate of time preference, respectively.

The budget constraint is given by

(11) $${\dot{W}}_{t}=r_{t}W_{t}+w_{t}\left(L_{Y,t}+L_{A,t}\right)-C_{t},$$

where $W_{t}$ is the household's financial asset. The time constraint is given by

(12) $$1=L_{Y,t}+L_{A,t}+l_{t}.$$

The household maximizes (11) by choosing a consumption stream and allocating time between leisure and the labor supply. The first-order conditions are

(13) $$\frac{\beta }{C_{t}}=\lambda_{t},$$

(14) $$\frac{1-\beta }{l_{t}}=w_{t}\lambda_{t},$$

(15) $$-{\dot{\lambda }}_{t}+\rho \lambda_{t}=r_{t}\lambda_{t},$$

(16) $$\lim_{T\to \infty }\lambda_{T}W_{T}e^{-\rho T}=0,$$

where $\lambda_{t}$ and (16) represent the shadow price of assets and the transversality condition, respectively. Substituting (13) into (14) yields

(17) $$w_{t}=\frac{1-\beta }{\beta }\frac{C_{t}}{l_{t}},$$

where (17) states that the wage rate equals the marginal substitution rate between consumption and leisure. Using (13) and (15) yields the following Euler equation:

(18) $$\frac{{\dot{C}}_{t}}{C_{t}}=r_{t}-\rho .$$

### Market

The economy is composed of pollution permits and labor, capital, stock, and goods markets. The pollution permits allocated by the government are equal to the pollution emitted by the final good firms in equilibrium $P_{t}=\bar{P}$. The labor market is cleared as $1=L_{Y,t}+L_{A,t}+l_{t}$. The equilibrium condition for the capital market is $\int_{0}^{A_{t}}x_{j,t}dj=K_{t}$, or, $A_{t}x_{t}=K_{t}$. The no-arbitrage equation is as follows:

(19) $$\frac{\pi_{t}+{\dot{P}}_{A,t}}{P_{A,t}}=r_{t}.$$

Finally, in the good market, the following holds:

(20) $$Y_{t}=C_{t}+{\dot{K}}_{t}+Z_{t}.$$

### Equilibrium

We define two jump variables ($y_{t}\equiv Y_{t}/K_{t}$, $z_{t}\equiv C_{t}/K_{t}$) and one state variable ($\omega_{t}\equiv K_{t}/A_{t}$) to derive the following dynamic system:

(21) $$\frac{{\dot{y}}_{t}}{y_{t}}=\frac{1-\alpha }{\alpha }\left\{\left(\frac{1}{\bar{P}}\right)+\alpha \delta y_{t}^{\frac{1}{1-\alpha }}\omega_{t}-\alpha^{2}y_{t}\right\},$$

(22) $$\frac{{\dot{\omega }}_{t}}{\omega_{t}}=y_{t}-z_{t}-\left(\frac{1}{\bar{P}}\right)-\delta \left\{1-y_{t}^{\frac{1}{1-\alpha }}\omega_{t}-\frac{1-\beta }{\beta \left(1-\alpha \right)}y_{t}^{\frac{\alpha }{1-\alpha }}\omega_{t}z_{t}\right\},$$

(23) $$\frac{{\dot{z}}_{t}}{z_{t}}=z_{t}-\left(1-\alpha^{2}\right)y_{t}-\rho .$$

Appendix A shows their derivations. Here, ${\dot{y}}_{t}={\dot{\omega }}_{t}={\dot{z}}_{t}=0$ determines the following steady state:

(24) $$\omega^{*}\left(\bar{P}\right)=\frac{\alpha^{2}y^{*}\left(\bar{P}\right)-1/\bar{P}}{\alpha \delta y^{*}{\left(\bar{P}\right)}^{\frac{1}{1-\alpha }}},$$

(25) $$z^{*}\left(\bar{P}\right)=\left(1-\alpha^{2}\right)y^{*}\left(\bar{P}\right)+\rho ,$$

(26) $$y^{*}\left(\bar{P}\right)=\frac{\beta }{2\alpha \left(1+\alpha \right)}\left[B\left(\bar{P}\right)+\sqrt{B{\left(\bar{P}\right)}^{2}+\frac{4\alpha \left(1+\alpha \right)}{\beta }D\left(\bar{P}\right)}\right],$$

where

$$B\left(\bar{P}\right)\equiv \delta +\frac{\beta -\alpha }{\beta \left(1-\alpha \right)}\rho +\frac{1+\alpha }{\alpha \beta \bar{P}},\;D\left(\bar{P}\right)\equiv \frac{\rho \left(1-\beta \right)}{\alpha \beta \left(1-\alpha \right)\bar{P}}.$$

We show the derivation of the steady state in Appendix B.

To analytically prove the existence and stability of the steady state, we assume the following parameter condition:

**Assumption 1 [The parameter condition]**

*We assume that* $\alpha^{2}/\left(1-\alpha \right)<1-\beta$ *,* $\hat{\delta }<\hat{\alpha }<\delta$ *,* $\hat{\eta }<\eta_{P}$ *, and* $0<P_{1}<\bar{P}$ *hold. Each parameter is defined as follows:*

$$\hat{\alpha }\equiv \frac{\left(1+\alpha \right)\left(1-\beta \right)}{\alpha \beta },\;\hat{\eta }\equiv \frac{2\alpha {\left(1+\alpha \right)}^{2}\left(1-\beta \right)}{\delta {\left(1-\alpha \right)}^{2}\left[\beta -\alpha +2\alpha \left(1-\beta \right)\right]},\;\;P_{1}\equiv \frac{\beta \left(1-\alpha^{2}\right)}{\rho \alpha \left(\beta -\alpha \right)}.$$

From assumption 1, we obtain the following proposition:

**Proposition 1 [The existence and stability of a steady state]**

*From assumption 1, the dynamic system in (*21*)–(*23*) has a unique steady state* ($y^{*},\;\omega^{*},\;z^{*}$), *which is locally saddle-point stable.*

Proof See Appendix B for the steady state. Please refer to [12] for proof of the stability of the dynamic system. ■

In the steady state, the leisure and the labor spent in each sector become

(27) $$l^{*}\left(\bar{P}\right)=\frac{1-\beta }{\alpha \beta \delta \left(1-\alpha \right)}\left(1-\alpha^{2}+\frac{\alpha^{2}\rho }{r^{*}\left(\bar{P}\right)+1/\bar{P}}\right)r^{*}\left(\bar{P}\right),$$

(28) $$L_{Y}^{*}\left(\bar{P}\right)=\frac{r^{*}\left(\bar{P}\right)}{\alpha \delta },$$

(29) $$L_{A}^{*}\left(\bar{P}\right)=\delta -\frac{r^{*}\left(\bar{P}\right)}{\alpha }-\frac{1-\beta }{\alpha \beta \left(1-\alpha \right)}\left(1-\alpha^{2}+\frac{\alpha^{2}\rho }{r^{*}\left(\bar{P}\right)+1/\bar{P}}\right)r^{*}\left(\bar{P}\right).$$

We show their derivations in Appendix C.

In the steady state, the interest rates become

(30) $$r^{*}\left(\bar{P}\right)=\frac{\alpha }{2\beta \left(1-\alpha^{2}\right)}\left[R_{1}\left(\bar{P}\right)+\sqrt{R_{1}^{2}\left(\bar{P}\right)+\frac{4\beta \left(1-\alpha^{2}\right)R_{2}\left(\bar{P}\right)}{\alpha }}\right],$$

$$R_{1}\left(\bar{P}\right)\equiv \beta \left(1-\alpha \right)\left(\rho +\delta \right)-\alpha \rho \left(1-\beta \right)-\frac{1-\alpha^{2}}{\alpha }\left(1-\beta +\frac{\beta }{\bar{P}}\right)>0,$$

$$R_{2}\left(\bar{P}\right)\equiv \frac{1}{\bar{P}}\left[\beta \left(1-\alpha \right)\left(\rho +\delta \right)-\frac{\left(1-\alpha^{2}\right)\left(1-\beta \right)}{\alpha }\right]>0.$$

We show their derivations in Appendix C.

In the steady state, the growth rate is given by

(31) $$g\left(\bar{P}\right)=r^{*}\left(\bar{P}\right)-\rho ,$$

(32) $$g\left(\bar{P}\right)=\delta -\frac{r^{*}\left(\bar{P}\right)}{\alpha }-\frac{1-\beta }{\alpha \beta \left(1-\alpha \right)}\left(1-\alpha^{2}+\frac{\alpha^{2}}{r^{*}\left(\bar{P}\right)+1/\bar{P}}\right)r^{*}\left(\bar{P}\right),$$

where we show the derivation of the growth rate in Appendix C.

### Comparative statics for the effect of an environmental policy on the steady state

In this section, we investigate analytically how changes in pollution permit levels affect each endogenous variable, growth rate, and welfare. For the results from the numerical analysis, see [12]. Differentiating (24)–(26), and (30) with respect to $\bar{P}$, we obtain the following proposition:

**Proposition 2 [The effect of an environmental policy on the steady state]**

*There exists*$\bar{P}$*such that a reduction in pollution permit levels decreases the interest rate. Then, the reduction in*$\bar{P}$*increases the output per capita* ($y^{*}$) *and consumption per capita* ($z^{*}$) *and decreases the capital per variety* ($\omega^{*}$) *and interest rate* ($r^{*}$).

Proof See Appendix D. ■

As discussed in Proposition 3, the reduction in P promotes R&D activity; therefore, a new variety is developed by the firm in the R&D sector. This implies that firms in the final goods sector can produce more output in a subsequent period because they can input more aggregate physical capital. Hence, the consumption of the final good increased. This leads to an increase in the output per capita ($y^{*}$) and consumption per capita ($z^{*}$). On the other hand, an increase in new variety reduces capital per variety ($\omega^{*}$). The reduction in $\bar{P}$ raises the dividend per stock; therefore, households substitute purchasing share assets for renting capital. A household's arbitrage activity leads to a reduction in the interest rate.

Using the result of proposition 2, we obtain the following proposition:

**Proposition 3 [The effect of an environmental policy on each endogenous variable]**

*While the reduction in pollution permit levels increases the marginal cost of intermediate goods* ($p^{*}+1/\bar{P}$)*, monopoly profit per average capital* ($\pi^{*}/x^{*}$)*, price of a new design per average capital* ($P_{A}^{*}/x^{*}$), *wage per capital* ($w^{*}/K^{*}$)*, and labor in the R&D sector* ($L_{A}^{*}$)*, a reduction in pollution permit levels decreases labor in the final goods sector* ($L_{Y}^{*}$) *and leisure* ($l^{*}$).

Proof See Appendix E. ■

The reduction in $\bar{P}$ increases the marginal cost of the abatement good; thus, the intermediate good price increases, which increases the monopoly profit per average capital and the dividend per stock. This leads to an increase in the price of a new design per unit of average capital; thus, the demand for the new design increases. The increasing demand raises the demand for labor in the R&D sector; therefore, the wage rate per capita in the sector increases. Thus, the household substitutes labor for leisure and supplies labor from the firm in the final goods sector to the firm in the R&D sector. Finally, a reduction in P promotes R&D activities and stimulates economic growth. We call this the substitution effect. From the above discussion, we obtain the following proposition.

**Proposition 4 [The effect of an environmental policy on growth rate]**

*There exists* $\bar{P}$ *such that the reduction in pollution permit levels increases the economic growth rate through the substitution effect.*

Proof See Appendix E. ■

### Comparative statics for the effect of environmental policy on welfare

We analytically investigate the effects of a pollution permit level reduction on welfare. Our welfare measure is given in (10). By substituting $C_{t}=z^{*}\left(\bar{P}\right)K_{0}e^{gt}$ and (31) into (10), we can rewrite (10) as

(33) $$U\left(\bar{P}\right)=\frac{\beta }{\rho }\log z^{*}\left(\bar{P}\right)+\left[\frac{\beta }{\rho }\log K_{0}+\frac{\beta g\left(\bar{P}\right)}{\rho^{2}}\right]+\frac{\left(1-\beta \right)}{\rho }\log l^{*}\left(\bar{P}\right)-\frac{\eta_{P}}{\rho }\log\bar{P}.$$

The first three terms show the indirect effect of a reduction in $\bar{P}$ on welfare level through consumption, growth rate, and leisure. The fourth term shows the direct effect of a reduction in $\bar{P}$ on the welfare level.

Differentiating (33) with respect to $\bar{P}$, we can obtain the following proposition:

**Proposition 5 [The effect of an environmental policy on welfare]**

*There exists* $\bar{P}$ *such that reduction in pollution permit levels monotonously increases the welfare level.*

Proof See Appendix F. ■

Intuitively, Proposition 5 shows that reducing pollution permits raises welfare levels due to decreased pollution and increased consumption per capita and growth rate. However, the reduction in $\bar{P}$ decreases at the welfare level because of a reduction in leisure ($l^{*}\left(\bar{P}\right)$). Under assumption 1, the positive effect of $\bar{P}$ on welfare dominates its negative effect; $\bar{P}$ on welfare, the welfare level monotonously increases as $\bar{P}$ deceases.

## Conclusion

Governments are actively introducing stringent environmental policies to promote sustainable development. However, public opinion often opposes these policies, leading to higher unemployment. It is necessary to show that environmental policies reduce pollution, promote economic growth, and improve economic welfare, to gain a public understanding. This study analyes the impact of emission quota reductions on pollution emissions, economic growth rates, and economic welfare by introducing emissions trading into an R&D-based growth model with endogenous labor supply. A reduction in emission quota leads to an increase in the wage rate, which implies an increase in the opportunity cost of leisure. Households then substitute leisure time for labor, which increases the labor supply to the R&D sector and promotes innovative activities. Through this substitution effect, the analytical results show that under certain parameter conditions and pollution reduction, it leads to higher economic growth rates and improved welfare.

This study has several policy implications. First, household leisure is important to the growth effects of environmental policies. In general, countries with strong preferences for leisure have more leisure time. In these countries, environmental policies are more likely to promote economic growth and reduce pollution if leisure activities are substituted for innovative activities. On the other hand, in countries with weaker leisure preferences, much leisure is devoted to working. Even with stricter environmental policies, the rate of economic growth may not be as high as expected. This difference may influence the advantages and disadvantages of environmental policies. Second, the growth effects of this environmental policy depend on labor migration and the opportunity cost of leisure through the wage rate. They would depend on the efficiency of the labor market. In other words, environmental policies significantly impact the economic growth rate in countries with labor markets where wage rates are elastic, and labor supply changes smoothly. However, in countries with labor markets where wage rates are rigid and price adjustment mechanisms do not work well, environmental policies may impact the economic growth rate less, as leisure-to-work and inter-sectoral labor mobility may not proceed as expected. The results of this study suggest that policymakers aiming for sustainable development need to bear in mind preferences for leisure and the efficiency of labor markets.

The analysis thus far has derived the equilibrium and then conducted a comparative statics analysis to determine the impact of environmental policy on each economic variable. However, this study did not perform a comparative dynamic analysis. This analysis is important because it reveals the impact of environmental policies on the transition from an initial point to a steady state and because such policies may contribute to economic stabilisation by speeding up the convergence to a steady state. The analytical approach used in this study can be applied to numerical analysis. Based on [16], a comparative dynamic analysis was performed with the numerical analysis of [18]. However, no comparative dynamic analysis of [15] has been performed. The methods used in this study allow for a comparative dynamic analysis. If this analysis is performed, it can also be applied to the comparative dynamic analysis of [20]. The research in [20] focuses on social status preferences, an important concept in behavioral economics. Hence, this research agenda provides new insights into behavioral, environmental economics. The research questions await analysis.

## CRediT authorship contribution statement

**Yoshihiro Hamaguchi:** Conceptualization, Methodology, Writing – original draft, Visualization, Investigation. **Miraj Ahmed Bhuiyan:** Writing – original draft, Writing – review & editing. **Muhammad Khalilur Rahman:** Writing – review & editing.

## Declaration of Competing Interest

The authors declare that they have no known competing financial interests or personal relationships that could have appeared to influence the work reported in this paper.

## Data Availability

No data was used for the research described in the article.

## Appendix

This appendix details the process of deriving the equilibria in the text and the proofs of the comparative statics analysis for each proposition.

Appendix A for the derivation of the dynamic system

Using (1) and $P_{t}=\bar{P}$, we rewrite (5) as follows:

(A.1) $$\frac{p_{t}^{e}}{Z_{t}}=\frac{Z_{t}}{\int_{0}^{A_{t}}x_{j,t}dj}=\frac{1}{\bar{P}},$$

which is used with (4) to derive the following:

(A.2) $$p_{t}=\alpha {\left(\frac{L_{Y,t}}{x_{j,t}}\right)}^{1-\alpha }-\frac{1}{\bar{P}},$$

which is substituted into (6) to yield the following:

(A.3) $$\alpha {\left(\frac{L_{Y,t}}{x_{j,t}}\right)}^{1-\alpha }-\frac{1}{\bar{P}}=\frac{1}{\alpha }\left(r_{t}+\frac{1-\alpha }{\bar{P}}\right)\Leftrightarrow \frac{r_{t}}{\alpha }=\alpha L_{Y,t}^{1-\alpha }{\left(\frac{A_{t}}{K_{t}}\right)}^{1-\alpha }-\frac{1}{\alpha \bar{P}}\Leftrightarrow r_{t}=\alpha^{2}y_{t}-\frac{1}{\bar{P}},$$

which is used with (7) to derive the following:

(A.4) $$\pi_{t}=\alpha \left(1-\alpha \right)y_{t}\omega_{t}.$$

We rewrite (3) as follows:

(A.5) $$w_{t}=\left(1-\alpha \right)\frac{Y_{t}}{L_{Y,t}},$$

which is substituted into (9) to derive the following:

(A.6) $$\delta P_{A,t}A_{t}=\left(1-\alpha \right)y_{t}\frac{K_{t}}{L_{Y,t}}.$$

Here, $Y_{t}=K_{t}^{\alpha }{\left(A_{t}L_{Y,t}\right)}^{1-\alpha }$ is rewritten as follows:

(A.7) $$y_{t}={\left(\frac{A_{t}L_{Y,t}}{K_{t}}\right)}^{1-\alpha }\Leftrightarrow y_{t}^{\frac{1}{1-\alpha }}\frac{1}{A_{t}}=\frac{L_{Y,t}}{K_{t}}\Leftrightarrow \frac{K_{t}}{L_{Y,t}}=y_{t}^{-\frac{1}{1-\alpha }}A_{t},$$

which is substituted into (A.6) to yield the following:

(A.8) $$\delta P_{A,t}A_{t}=\left(1-\alpha \right)y_{t}^{1-\frac{1}{1-\alpha }}A_{t}\Leftrightarrow y_{t}={\left[\left(1-\alpha \right){\left(\delta P_{A,t}\right)}^{-1}\right]}^{\frac{1-\alpha }{\alpha }}.$$

Using $\omega_{t}=\left(K_{t}\right)/\left(A_{t}\right)$, we can rewrite $y_{t}={\left[\left(A_{t}L_{Y,t}\right)/\left(K_{t}\right)\right]}^{1-\alpha }$ as follows:

(A.9) $$y_{t}={\left(\frac{L_{Y,t}}{\omega_{t}}\right)}^{1-\alpha }\Leftrightarrow L_{Y,t}=y_{t}^{\frac{1}{1-\alpha }}\omega_{t}.$$

Next, substituting A.3) and (A.4) into ((19) yields the following:

(A.10) $$\frac{{\dot{P}}_{A,t}}{P_{A,t}}=r_{t}-\frac{\pi_{t}}{P_{A,t}}\Leftrightarrow \frac{{\dot{P}}_{A,t}}{P_{A,t}}=\alpha^{2}y_{t}-\left(\frac{1}{\bar{P}}\right)-\frac{\alpha \left(1-\alpha \right)y_{t}\omega_{t}}{P_{A,t}},$$

which is substituted into (A.10) to derive the following:

(A.11) $$\frac{{\dot{P}}_{A,t}}{P_{A,t}}=\alpha^{2}y_{t}-\left(\frac{1}{\bar{P}}\right)-\alpha \delta y_{t}^{\frac{1}{1-\alpha }}\omega_{t}.$$

Furthermore, using the time derivative of (A.8) yields the following:

(A.12) $$\frac{\alpha }{1-\alpha }\frac{{\dot{y}}_{t}}{y_{t}}=-\frac{{\dot{P}}_{A,t}}{P_{A,t}},$$

which is used with A.11) to derive ((21).

Using (6) and $P_{t}=\bar{P}$, we can rewrite (20) as follows:

(A.13) $$\frac{{\dot{K}}_{t}}{K_{t}}=y_{t}-z_{t}-\left(\frac{1}{\bar{P}}\right).$$

Substituting (9) into (17) yields the following:

(A.14) $$\delta P_{A,t}A_{t}=\frac{1-\beta }{\beta }\frac{C_{t}}{l_{t}}\Leftrightarrow l_{t}=\frac{1-\beta }{\beta \delta }\frac{C_{t}}{K_{t}}\frac{K_{t}}{A_{t}}\frac{1}{P_{A,t}}\Leftrightarrow l_{t}=\frac{1-\beta }{\beta \left(1-\alpha \right)}y_{t}^{\frac{\alpha }{1-\alpha }}\omega_{t}z_{t},$$

which is used with (12) and A.7) to rewrite ((8) as follows:

(A.15) $$\frac{{\dot{A}}_{t}}{A_{t}}=\delta \left(1-y_{t}^{\frac{1}{1-\alpha }}\omega_{t}-\frac{1-\beta }{\beta \left(1-\alpha \right)}y_{t}^{\frac{\alpha }{1-\alpha }}\omega_{t}z_{t}\right),$$

which is used with A.13) to derive ((22).

Substituting A.3) into ((18), we obtain the following:

(A.16) $$\frac{{\dot{C}}_{t}}{C_{t}}=\alpha^{2}y_{t}-\left(\frac{1}{\bar{P}}\right)-\rho ,$$

which is used with A.13) to yield ((23).

## Appendix B for the derivation of the steady state

Here, ${\dot{y}}_{t}={\dot{\omega }}_{t}={\dot{z}}_{t}=0$ determines the steady state. Then, (21) and (23) lead to (24) and (25). Substituting (24) and (25) into (22) yields the following quadratic equation of $y^{*}\left(\bar{P}\right)$:

(B.1) $$f\left(y^{*}\left(\bar{P}\right)\right)\equiv \frac{\alpha \left(1+\alpha \right)}{\beta }y^{*}{\left(\bar{P}\right)}^{2}-B\left(\bar{P}\right)y^{*}\left(\bar{P}\right)-D\left(\bar{P}\right)=0,$$

for

$$B\left(\bar{P}\right)\equiv \left[\delta +\frac{\left(\beta -\alpha \right)\rho }{\beta \left(1-\alpha \right)}+\frac{1+\alpha }{\alpha \beta \bar{P}}\right],D\left(\bar{P}\right)\equiv \frac{\rho \left(1-\beta \right)}{\alpha \beta \left(1-\alpha \right)\bar{P}},$$

where solving equation B.1) yields a positive solution $\left(y^{*}\left(\bar{P}\right)>0\right)$ and a negative solution $\left(y^{*}\left(\bar{P}\right)<0\right)$. We choose the positive solution $\left(y^{*}\left(\bar{P}\right)>0\right)$ as the solution to (B.1). Then, we obtain ((26).

## Appendix C for the derivation of labor, leisure, interest rates, and growth rate

First, we derive labor and leisure in a steady state. Using A.3) and ((24) yields the following:

(C.1) $$\omega^{*}\left(\bar{P}\right)=\frac{r^{*}\left(\bar{P},\theta \right)}{\alpha \delta y^{*}{\left(\bar{P}\right)}^{\frac{1}{1-\alpha }}},$$

which is used in A.3) and (A.9) to derive ((28).

Substituting (25) and (C.1) into (A.14) yields the following:

(C.2) $$l^{*}\left(\bar{P}\right)=\frac{r^{*}\left(1-\beta \right)}{\alpha \beta \delta \left(1-\alpha \right)}\left(1-\alpha^{2}+\frac{\rho }{y^{*}\left(\bar{P}\right)}\right),$$

which is used in A.3) to derive ((27). Substituting (28) and (27) into (12), we obtain (29).

Second, we derive the interest rate in a steady state. Using (31) and (32) yields the following quadratic equation for $r^{*}\left(\bar{P}\right)$:

(C.3) $$h\left(r^{*}\left(\bar{P}\right)\right)\equiv \frac{\beta \left(1-\alpha^{2}\right)}{\alpha }r^{*}{\left(\bar{P}\right)}^{2}-R_{1}\left(\bar{P}\right)r^{*}\left(\bar{P}\right)-R_{2}\left(\bar{P}\right)=0,$$

if

$$R_{1}\left(\bar{P}\right)>0\Leftrightarrow \left[\beta \delta \left(1-\alpha \right)-\frac{\left(1-\alpha^{2}\right)\left(1-\beta \right)}{\alpha }\right]+\left[\rho \left(\beta -\alpha \right)-\frac{\beta \left(1-\alpha^{2}\right)}{\alpha \bar{P}}\right]>0,$$

and

(C.4) $$\delta >\frac{\left(1+\alpha \right)\left(1-\beta \right)}{\alpha \beta }\equiv \hat{\alpha },$$

(C.5) $$\bar{P}>\frac{\beta \left(1-\alpha^{2}\right)}{\alpha \rho \left(\beta -\alpha \right)}\equiv P_{1},$$

(C.6) $$R_{2}\left(\bar{P}\right)>0\Leftrightarrow \rho +\delta >\hat{\alpha },$$

where C.3) has a positive solution $\left(r^{*}\left(\bar{P}\right)>0\right)$ and a negative solution $\left(r^{*}\left(\bar{P}\right)<0\right)$. Subsequently, ((30) is derived by choosing $r^{*}\left(\bar{P}\right)>0$ as the solution of (C.3).

Finally, the growth rate in the steady state is derived as follows: (31) is obtained using (18) and A.3) while ((32) are obtained by using (8), (12), (28), and (27).

## Appendix D for proof of proposition 2

First, we prove the effect of $\bar{P}$ on the interest rate. Differentiating (C.3) with respect to $\bar{P}$ yields the following:

(D.1) $$\begin{array}{cc} & \frac{\partial h\left(r^{*}\left(\bar{P}\right)\right)}{\partial r^{*}\left(\bar{P}\right)}\frac{\partial r^{*}\left(\bar{P}\right)}{\partial \bar{P}}=\left[\frac{2\beta \left(1-\alpha^{2}\right)}{\alpha }-R_{1}\left(\bar{P}\right)\right]\frac{\partial r^{*}}{\partial \bar{P}}-\frac{\beta \left(1-\alpha^{2}\right)}{\alpha \bar{P}}r^{*}\left(\bar{P}\right)\\ & \;+\;\frac{1}{{\bar{P}}^{2}}\left[\beta \left(1-\alpha \right)\left(\rho +\delta \right)-\frac{\left(1-\alpha^{2}\right)\left(1-\beta \right)}{\alpha }\right]=0, \end{array}$$

where is rewritten as follows:

$$\frac{\partial r^{*}\left(\bar{P}\right)}{\partial \bar{P}}=\frac{\alpha^{-1}\beta \left(1-\alpha^{2}\right)-\beta \left(1-\alpha \right)\left[\rho +\delta -{\left(\alpha \beta \right)}^{-1}\left(1+\alpha \right)\left(1-\beta \right)\right]}{{\bar{P}}^{2}\sqrt{R_{1}^{2}\left(\bar{P}\right)+4\alpha^{-1}\beta \left(1-\alpha^{2}\right)R_{2}\left(\bar{P}\right)}},$$

which is represented as follows:

(D.2) $$\frac{\partial r^{*}}{\partial \bar{P}}\overset{\geq }{<}0,\;\text{if}\;r^{*}\overset{\geq }{<}\frac{\alpha \left(\rho +\delta -\hat{\alpha }\right)}{1+\alpha }\equiv {\hat{r}}_{P},$$

which is further substituted into (C.3) to derive $h(\hat{r_{P}})=0$. Solving for $h(\hat{r_{P}})=0$ yields the following:

(D.3) $$\bar{P}=\frac{\beta \left(1-\alpha \right)\left[\alpha^{-1}\left(1+\alpha \right){\hat{r}}_{P}-\hat{\alpha }\right]}{{\hat{r}}_{P}\left[\beta \left(1-\alpha \right)\left(\rho +\delta -\hat{\alpha }\right)-\alpha \rho \left(1-\beta \right)-\alpha^{-1}\beta \left(1-\alpha^{2}\right){\hat{r}}_{P}\right]-\alpha \left(1-\beta \right)},$$

where $r^{*}={\hat{r}}_{P}$ of (D.2) is substituted into (D.3) to derive $\bar{P}=0$. Hence, we obtain the following:

(D.4) $$\frac{\partial r^{*}\left(\bar{P}\right)}{\partial \bar{P}}>0,\;\text{if}\;0<P_{1}<\bar{P}.$$

Using equation (D.4), we can prove the effect of $\bar{P}$ on the steady state. Taking the total differential (B.1) with respect to $\bar{P}$ yields

(D.5) $$\frac{\partial f\left(y^{*}\left(\bar{P}\right)\right)}{\partial \bar{P}}=\left[\frac{2\alpha \left(1+\alpha \right)y^{*}\left(\bar{P}\right)}{\beta }-R_{1}\left(\bar{P}\right)\right]\frac{\partial y^{*}}{\partial \bar{P}}+\frac{\left(1+\alpha \right)}{\alpha \beta {\bar{P}}^{2}}y^{*}\left(\bar{P}\right)+\frac{R_{2}\left(\bar{P}\right)}{\bar{P}}=0,$$

which is rewritten with assumption 1 as follows:

(D.6) $$\frac{\partial y^{*}\left(\bar{P}\right)}{\partial \bar{P}}=-\frac{1}{\alpha \beta {\bar{P}}^{2}}{\left(\sqrt{R_{1}^{2}\left(\bar{P}\right)+\frac{4\alpha \left(1+\alpha \right)}{\beta }R_{2}\left(\bar{P}\right)}\right)}^{-1}\left[\left(1+\alpha \right)y^{*}\left(\bar{P}\right)+\frac{\rho \left(1+\beta \right)}{1-\alpha }\right]<0.$$

Differentiating (25) with respect to $\bar{P}$ and using (D.6), we obtain

(D.7) $$\frac{\partial z^{*}\left(\bar{P}\right)}{\partial \bar{P}}=\left(1+\alpha \right)\frac{\partial y^{*}\left(\bar{P}\right)}{\partial \bar{P}}<0.$$

Differentiating (C.1) with respect to $\bar{P}$ and, using (D.6) and (D.7), we obtain the following:

(D.8) $$\frac{\partial \omega^{*}\left(\bar{P}\right)}{\partial \bar{P}}=\frac{1}{\alpha \delta y^{*}}\underset{\left(+\right)}{\underset{︸}{\frac{\partial r^{*}\left(\bar{P}\right)}{\partial \bar{P}}}}-\frac{1}{\alpha \delta y^{*2-\alpha }}\underset{\left(-\right)}{\underset{︸}{\frac{\partial y^{*}\left(\bar{P}\right)}{\partial \bar{P}}}}>0.$$

## Appendix E for proof of propositions 3 and 4

First, we prove Proposition 3: Using (A.2) and (A.3) yields the following:

(E.1) $$p^{*}+\frac{1}{\bar{P}}=\alpha y^{*}\Rightarrow \frac{\partial \left[p^{*}+1/\bar{P}\right]}{\partial \bar{P}}=\alpha \underset{-}{\underset{︸}{\frac{\partial y^{*}\left(\bar{P}\right)}{\partial \bar{P}}}}<0.$$

Combing (6) and (7) with (E.1) yields

(E.2) $$\frac{\pi^{*}}{x^{*}}=\left(p^{*}+\frac{1}{\bar{P}}\right)\Rightarrow \frac{\partial \left[\pi^{*}/x^{*}\right]}{\partial \bar{P}}=\left(1-\alpha \right)\frac{\partial \left[p^{*}+1/\bar{P}\right]}{\partial \bar{P}}=\alpha \left(1-\alpha \right)\underset{-}{\underset{︸}{\frac{\partial y^{*}\left(\bar{P}\right)}{\partial \bar{P}}}}<0.$$

Differentiating the rewritten (A.10) with respect to $\bar{P}$ and using (D.5) yields the following:

(E.3) $$\frac{\partial r^{*}}{\partial \bar{P}}\overset{\geq }{<}0,\;\text{if}\;r^{*}\overset{\geq }{<}\frac{\alpha \left(\rho +\delta -\hat{\alpha }\right)}{1+\alpha }\equiv {\hat{r}}_{P},$$

Differentiating (9) with respect to $\bar{P}$ and using (E.3) yields the following:

(E.4) $$\frac{\partial \left[w^{*}/K^{*}\right]}{\partial \bar{P}}=\delta \underset{-}{\underset{︸}{\frac{\partial \left[P_{A}^{*}/x^{*}\right]}{\partial \bar{P}}}}<0.$$

Differentiating (28) with respect to $\bar{P}$ yields the following:

(E.5) $$\frac{\partial L_{Y}^{*}}{\partial \bar{P}}=\frac{1}{\alpha \delta }\underset{+}{\underset{︸}{\frac{\partial r^{*}\left(\bar{P}\right)}{\partial \bar{P}}}}>0.$$

Second, we prove proposition 4. Differentiating (C.2) with respect to $\bar{P}$ yields the following:

(E.6) $$\frac{\partial l^{*}\left(\bar{P}\right)}{\partial \bar{P}}=\frac{1-\beta }{\alpha \beta \delta \left(1-\alpha \right)}\left(1-\alpha^{2}+\frac{\rho }{y^{*}\left(\bar{P}\right)}\right)\left[\underset{+}{\underset{︸}{\frac{\partial r^{*}\left(\bar{P}\right)}{\partial \bar{P}}}}-\frac{r^{*}\left(\bar{P}\right)\rho }{y^{*}{\left(\bar{P}\right)}^{2}}\underset{-}{\underset{︸}{\frac{\partial y^{*}\left(\bar{P}\right)}{\partial \bar{P}}}}\right]>0,$$

which (D.6) is used to derive. Hence, we obtain $\partial l^{*}/\partial \bar{P}>0$ when (E.6) holds true.

Finally, Proposition 5 is proved. Using (32), (D.5), and (E.6), we obtain

$$\frac{\partial g\left(\bar{P}\right)}{\partial \bar{P}}=-\frac{1}{\alpha }\underset{+}{\underset{︸}{\frac{\partial r^{*}\left(\bar{P}\right)}{\partial \bar{P}}}}-\delta \underset{+}{\underset{︸}{\frac{\partial l^{*}\left(\bar{P}\right)}{\partial \bar{P}}}}<0.$$

## Appendix F for proof of propositions 5

We prove proposition 5. Differentiating (33) with respect to $\bar{P}$ yields the following:

(F.1) $$\frac{\partial U\left(\bar{P}\right)}{\partial \bar{P}}=\left[\frac{\beta }{\rho z^{*}}\frac{\partial z^{*}\left(\bar{P}\right)}{\partial \bar{P}}+\frac{\beta }{\rho^{2}}\frac{\partial g\left(\bar{P}\right)}{\partial \bar{P}}\right]-\left[\frac{\eta_{P}}{\rho \bar{P}}-\frac{1-\beta }{\rho l^{*}}\frac{\partial l^{*}\left(\bar{P}\right)}{\partial \bar{P}}\right]<0,\;\mathrm{if}\;E\left(\bar{P}\right)\equiv \left[\frac{\eta_{P}}{\rho \bar{P}}-\left(\frac{1-\beta }{\rho l^{*}}\right)l_{1}\right]>0,$$

which is rewritten with (E.7) as follows:

$$E\left(\bar{P}\right)=-\left(\frac{1-\beta }{\rho l^{*}\left(\bar{P}\right)}\right)\left[\frac{\alpha \left(1-\beta \right)}{\beta \delta \left(1-\alpha \right){\left(r^{*}\left(\bar{P}\right)+{\bar{P}}^{-1}\right)}^{2}}\right]\frac{\partial r^{*}\left(\bar{P}\right)}{\partial \bar{P}}+F\left(\bar{P}\right)<0,$$

if

(F.2) $$F\left(\bar{P}\right)\equiv \left[\frac{\alpha \rho {\left(1-\beta \right)}^{2}}{\beta \delta \rho {\bar{P}}^{2}l^{*}\left(\bar{P}\right)\left(1-\alpha \right){\left(r^{*}+{\bar{P}}^{-1}\right)}^{2}}-\frac{\eta_{P}}{\rho \bar{P}}\right]<0,$$

which is further rewritten with (27) and (A.3) as the following quadratic equation of y.

(F.3) $$F\left(\bar{P}\right)<0\Leftrightarrow u\left(y\right)\equiv \left(1-\alpha^{2}\right)y^{2}+y-\frac{\rho \left(1-\beta \right)}{\alpha^{2}\delta \eta_{P}\bar{P}}>0,$$

where solving (F.3) leads to derve a positive solution (y>0) and a negative solution (y<0). Subsequently, we obtain the following positive solution (y>0):

(F.4) $$y_{u}\equiv \frac{1}{2\left(1-\alpha^{2}\right)}\left[-1+\sqrt{1+\frac{4\rho \left(1-\alpha^{2}\right)\left(1-\beta \right)}{\alpha^{2}\delta \eta_{P}\bar{P}}}\right].$$

Thus, we can obtain $\partial U\left(\bar{P}\right)/\partial \bar{P}$ when $0<y_{u}<y^{*}\left(\bar{P}\right)$ holds.

We prove $0<y_{u}<y^{*}\left(\bar{P}\right)$. Using (26) and (F.4) yields the followings:

$$y^{*}\left(\bar{P}\right)-y_{u}=\frac{\beta B\left(\bar{P}\right)}{2\alpha \left(1+\alpha \right)}+\frac{1}{\left(1-\alpha^{2}\right)}+H\left(\bar{P}\right)>0,$$

if

(F.5) $$H\left(\bar{P}\right)\equiv \frac{\beta }{2\alpha \left(1+\alpha \right)}\sqrt{B{\left(\bar{P}\right)}^{2}+\frac{4\alpha \left(1+\alpha \right)}{\beta }D\left(\bar{P}\right)}-\frac{1}{2\left(1-\alpha^{2}\right)}\sqrt{1+\frac{4\rho \left(1-\alpha^{2}\right)\left(1-\beta \right)}{\alpha^{2}\delta \eta_{P}\bar{P}}}>0,$$

which is rewritten as follows:

$$\begin{array}{c} 2\delta {\left[\frac{\rho \left(\beta -\alpha \right)}{\beta \left(1-\alpha \right)}+\frac{1+\alpha }{\alpha \beta \bar{P}}\right]}^{2}+{\left(\frac{1+\alpha }{\alpha \beta \bar{P}}\right)}^{2}+{\left[\frac{\rho \left(\beta -\alpha \right)}{\beta \left(1-\alpha \right)}\right]}^{2}+\\ \left[\delta^{2}-{\left(\frac{\alpha \left(1+\alpha \right)}{\beta {\left(1-\alpha \right)}^{2}}\right)}^{2}\right]+\left[\frac{2\rho \left(1+\alpha \right)\left[\left(\beta -\alpha \right)+2\alpha \left(1-\beta \right)\right]}{\alpha \beta^{2}\left(1-\alpha \right)\bar{P}}-\frac{4\rho \left(1-\alpha^{2}\right){\left(1+\alpha \right)}^{2}\left(1-\beta \right)}{\delta \beta^{2}\eta_{P}\bar{P}{\left(1-\alpha \right)}^{4}}\right]>0, \end{array}$$

if

(F.6) $$\delta^{2}>{\left(\frac{\alpha \left(1+\alpha \right)}{\beta {\left(1-\alpha \right)}^{2}}\right)}^{2}\Leftrightarrow \delta >\frac{\alpha \left(1+\alpha \right)}{\beta \left(1-\alpha \right)}\equiv \hat{\delta },$$

and

$$\frac{2\rho \left(1+\alpha \right)\left[\left(\beta -\alpha \right)+2\alpha \left(1-\beta \right)\right]}{\alpha \beta^{2}\left(1-\alpha \right)\bar{P}}>\frac{4\rho \left(1-\alpha^{2}\right){\left(1+\alpha \right)}^{2}\left(1-\beta \right)}{\delta \beta^{2}\eta_{P}\bar{P}{\left(1-\alpha \right)}^{4}}$$

which is further rewritten as follows:

(F.7) $$\eta_{P}>\frac{2\alpha {\left(1+\alpha \right)}^{2}\left(1-\beta \right)}{\delta {\left(1-\alpha \right)}^{2}\left[\beta -\alpha +2\alpha \left(1-\beta \right)\right]}\equiv \hat{\eta }.$$

Thus, we can obtain $\partial U\left(\bar{P}\right)/\partial \bar{P}$ when (F.6) and (F.7) hold true.

Using (C.4) and (F.7), we can obtain the following:

$$\hat{\alpha }>\hat{\delta }\Leftrightarrow 1-\beta >\frac{\alpha^{2}}{1-\alpha }.$$
